# Identification of *Aspergillus fumigatus* UDP-Galactopyranose Mutase Inhibitors

**DOI:** 10.1038/s41598-017-11022-5

**Published:** 2017-09-07

**Authors:** Julia S. Martin del Campo, Meital Eckshtain-Levi, Nancy J. Vogelaar, Pablo Sobrado

**Affiliations:** 1 Department of Biochemistry, Virginia Tech, Blacksburg, VA 24061 USA; 2 Virginia Tech Center for Drug Discovery, Virginia Tech, Blacksburg, VA 24061 USA

## Abstract

*Aspergillus fumigatus* is an opportunistic human pathogen responsible for deadly, invasive infections in immunocompromised patients. The *A. fumigatus* cell wall is a complex network of polysaccharides among them galactofuran, which is absent in humans. UDP-galactopyranose mutase (UGM) catalyzes the conversion of UDP-galactofuranose (UDP-Gal*f*) to UDP-galactopyranose (UDP-Gal*p*) and is an important virulence factor. UGM is a flavin-dependent enzyme that requires the reduced flavin for activity; flavin reduction is achieved by reaction with NADPH. The aim of this work was to discover inhibitors of UGM by targeting the NADPH binding site using an ADP-TAMRA probe in a high-throughput screening assay. The flavonoids (2*S*)-hesperetin and (2*S*)-naringenin were validated as competitive inhibitors of UGM against NADPH with K_i_ values of 6 µM and 74 µM, respectively. To gain insight into the active chemical substituents involved in the inhibition of UGM, several derivatives of these inhibitors were studied. The results show that the hydroxyl groups of (2*S*)-hesperetin are important for inhibition, in particular the phenyl-chroman moiety. Congo red susceptibility assay and growth temperature effects showed that these compounds affected cell wall biosynthesis in *A. fumigatus*. This work is the first report of inhibition studies on UGM from eukaryotic human pathogens.

## Introduction

**Figure 1:**
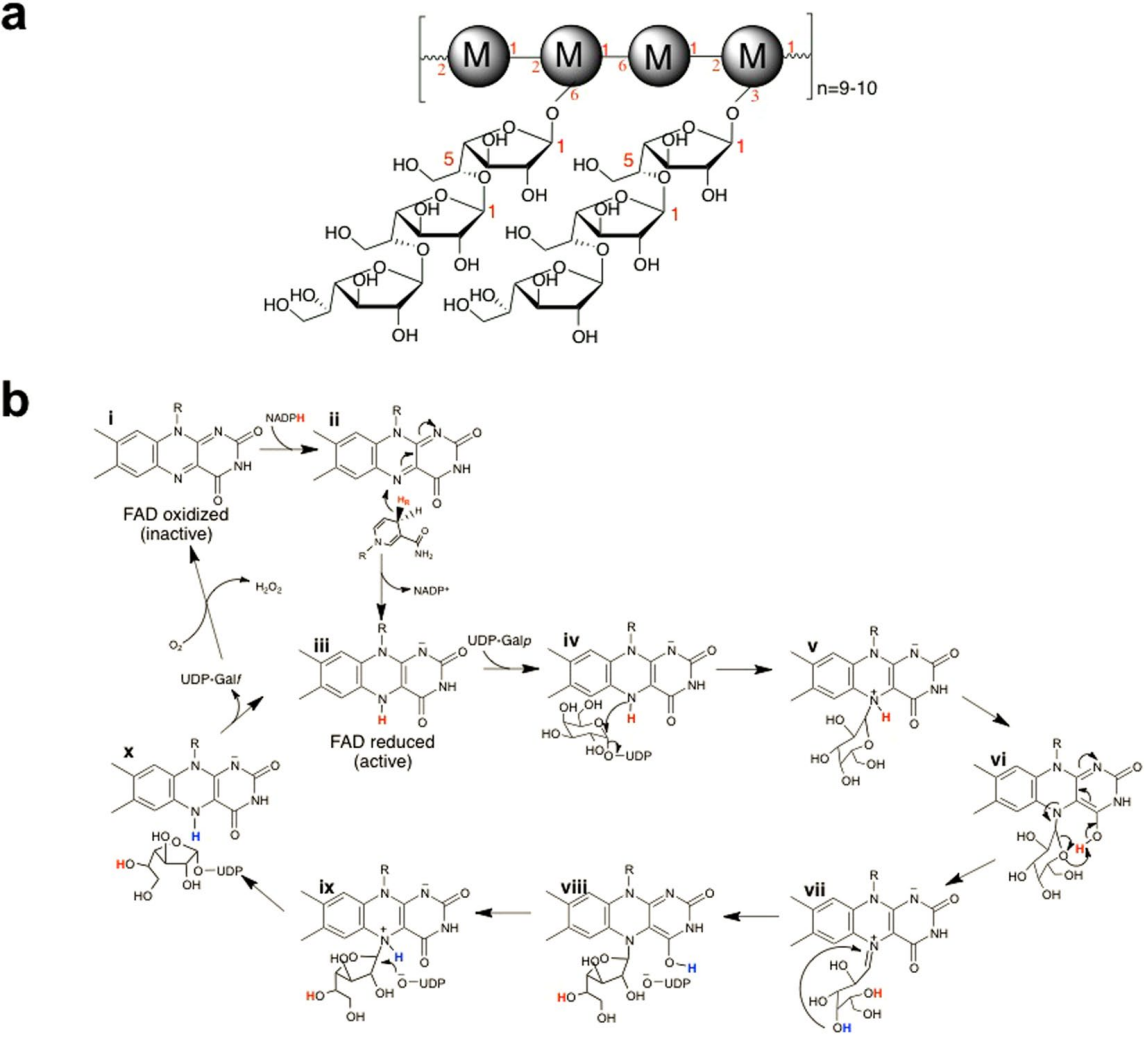
**(a)** Depiction of secreted galactomannan showing the (1 → 5)-linked Gal*f* chains bound to mannose units (M). **(b)** Chemical mechanism for UGM. The activation of UGM by NADPH is depicted in i and ii. The reduced activated enzyme (iii), binds to UDP-Galp and a covalent flavin–galactose adduct is formed via the direct attack of the FAD-N5﻿ atom to the Galp-C1 atom. This step leads to cleavage of the anomeric bond (iv-v). Tautomerization of the flavin permits the transfer of the FAD-N5-proton (shown in red) to the C4FAD = O (vi). This proton is next transferred to the Galp-C5-O, facilitating the opening of the sugar ring and formation of the flavin iminium ion (vi-vii). The FAD-C4 = O is predicted to accept the proton from the Galp-C4-OH (shown in blue) during ring contraction (viii). The final step is the direct attack of UDP to the FAD–galactofuranose adduct (ix-x).

*Aspergillus fumigatus* is a common opportunistic human pathogen that causes infections such as allergic bronchopulmonary aspergillosis and invasive pulmonary aspergillosis, among others^1,2^. Patients with acute leukemia, hematopoietic stem cell transplant recipients, and solid-organ transplant recipients are the three most common groups of patients at risk of invasive *A. fumigatus* infections^3^. Although drugs are available for treating *A. fumigatus* infections, the mortality rate among immunocompromised persons is >50%^4^. The fungal cell wall is essential to maintaining cell integrity and plays an important role in host-pathogen interactions. Several cell wall components are fungal specific and absent in mammals^5^. The cell wall of *A. fumigatus* consists of a three-dimensional arrangement of polysaccharides. The central core is composed of branched β−1,3-glucan cross-linked to chitin. The external core of the cell wall is composed of glucose chains of β-1,3–1,4 glucan and galactomannan, which makes the structure unique compared to other fungi^6^. Galactomannans are the major antigens produced during infection by *A. fumigatus*^7^ and possibly act as components of extracellular adhesive structures during host tissue invasion^8^. Secreted galactomannan is composed of a branched core containing α(1 → 2)- and α(1 → 6)-linked mannose, with β(1 → 5)-galactofuranose^9^ and/or β(1 → 4)-galactopyranose moieties linked linearly in side chains terminated by galactofuranose non-reducing end units^7,10,11^ (Fig. 1a). Galactofuranose (Gal*f*) is a five-member cyclic hexose found in several pathogens but is absent in humans. The synthesis of Gal*f* starts in the cytosol where UDP-galactopyranose (UDP-Gal*p*) is transformed to UDP-Gal*f* by UDP-galactofuranose mutase (UGM, Fig. 1b).

UGM is a key enzyme in the biosynthesis of galactofuranose containing glyco-conjugates. Deletion of *A. fumigatus* UGM prevents Gal*f* production and results in a strain with reduced cell wall thickness and attenuated virulence in mice^12^. In addition, UGM has been shown to be essential for the pathogenesis of *Mycobacterium tuberculosis*^13^ and for larval hatching and long-term survival of the nematodes *Brugia malayi*^14,15^ and *Caenorhabditis elegant*^16^. In the protozoan pathogen *Leishmania major*, UGM has also been identified as a virulence factor^17^. UGM is a flavoenzyme that catalyzes a non-redox reaction; however, the flavin is required to be in the reduced form for catalysis^18^. The catalytic cycle of UGM starts with NADPH binding, then reduction of the FAD occurs and, finally, release of NADP^+^ (Fig. 1b,i–iii). UDP-Gal*p* binds to the reduced UGM (Fig. 1b,iv) and the chemical steps are initiated by nucleophilic attack of the FAD N5 atom to the Gal*p* C1 atom, generating a flavin-Gal*p* intermediate (Fig. 1b,iv–v). Formation of a flavin-sugar iminium ion leads to opening of the sugar ring (Fig. 1b,vi–vii). After recyclization, attack by UDP releases the sugar of the flavin and forms the product, UDP-Gal*f* (Fig. 1b,viii–x)^19^. UGMs can react with molecular oxygen leading to the oxidation of the flavin, which is the inactive form of the enzyme (Fig. 1b,i). FAD oxidation occurs very slowly after following several hundred mutase reactions^20^.

Crystallographic data showed that the ADP group of NAD(P)H and the UDP group of the substrate have independent binding pockets, while the nicotinamide riboside binding site overlaps with the binding of the Gal*p* moiety in order to access the flavin N5 atom^19,21^. The unique arrangement of the NAD(P)H binding domain of UGM represents an attractive target for the design of inhibitors of the oxidized (inactive) enzyme that bind in the pocket, which is unique to the ADP component of NADPH.

In this work, a 2320 compound library was screened against oxidized *A. fumigatus* UGM using a TAMRA labeled ADP (ADP-TAMRA)^22^. The flavonoids (2*S*)-hesperetin and (2*S*)-naringenin were identified as competitive inhibitors against NADPH. Binding of inhibitors caused an increase in the melting temperature of UGM (~2.7 °C). Inhibition studies of (2*S*)-hesperetin derivatives allowed us to identify the hydroxyl group of the phenyl-chroman moiety as essential for binding and inhibition. In cultures of *A. fumigatus*, (2*S*)-hesperetin and (2*S*)-naringenin increased thermosensitivity and susceptibility to the cell wall stressing agent congo red. This work represents the first efforts in inhibitor discovery against eukaryotic UGM.

## Results

### High-throughput screening assay and hit validation

Previously, our group developed an ADP-TAMRA probe to identify inhibitors of flavin-dependent monooxygenases that use NADPH as a substrate^22^. To determine the utility of ADP-TAMRA to search for inhibitors of recombinant *A. fumigatus* UGM (UGM, from here on), ADP-TAMRA^22^ binding was tested as a function of UGM concentration. The observed increase in the anisotropy as a function of UGM concentration permitted the calculation of a K_D_ value of 6.7 µM (Fig. S1a). Competitive binding of ADP or NADP^+^ against ADP-TAMRA (Fig. S1b) was confirmed from the K_D_ value of the UGM-chromophore complex (anisotropy value of ~0.18) upon addition of increasing concentrations of either ligand. Thus, the change in anisotropy, upon release of ADP-TAMRA from the active site of UGM was used as the signal for a high-throughput screening (HTS) assay to identify small compounds that bound to UGM. Using this HTS assay, the Spectrum Collection library (2320 compounds) was screened against UGM at 20 µM concentration (2% DMSO) as described in the methods section. The Spectrum library was selected because of its diverse composition of bioactive compounds (60%), natural compounds of unknown biological properties (25%), and compounds representative of known drug-enzyme inhibitors, among others.

**Figure 2:**
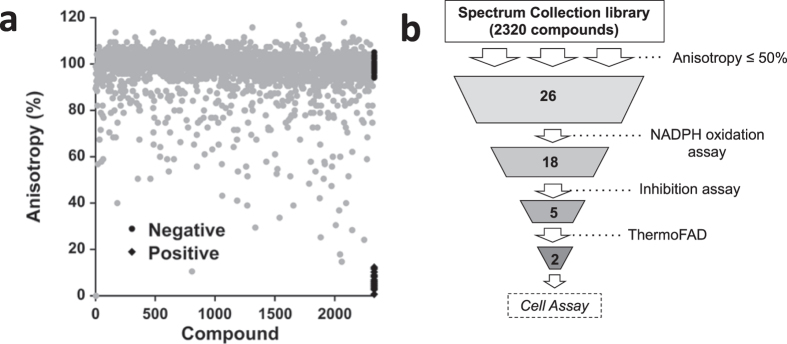
**(a)** HTS against UGM using ADP-TAMRA (30 nM). **(b)** Funnel diagram illustrating the processes of inhibitor identification and validation.

It is known that aggregate-based inhibition is time dependent^23^. In order to remove aggregators from screening, we measured the anisotropy after incubation at 30 min and 120 min. The difference between the two-time point readings was used to assess aggregation. Compounds with absolute difference in anisotropy between readings that was greater than 12% (corresponding to 3 standard deviations of the negative control) were considered aggregators^24^ and not studied further, leaving 2130 compounds (Fig. 2a). The average Z′ factor was calculated to be 0.82 ± 0.04, which indicates a wide separation between positive and negative controls.

Hits were defined as compounds that had an anisotropy ≤50% at 30 min incubation (Fig. 2b). From the 26 identified hits, compounds that oxidized NADPH were removed. Only 13 compounds were commercially available and they were classified as flavonoids and non-flavonoids (Table S1). Inhibition of product formation followed by UPLC at 20 and 200 µM compound concentration in 2% DMSO allowed for the identification of five compounds that exhibited a concentration dependent potency: (2*S*)-hesperetin, (2*S*)-naringenin, and chrysin from the flavonoid family, and diclazuril and febuxostat from the non-flavonoid family. The melting temperature of UGM (measured with the ThermoFAD method)^25^ with 20, 100, and 1000 µM of compound (2% DMSO and 1 mg/mL UGM) indicated that diclazuril and febuxostat are UGM denaturants, as these compounds reduced the T_m_ compared to the reference (UGM alone in 2% DMSO) and were not further studied. Chrysin showed no effect on T_m_ and (2*S*)-hesperetin and (2*S*)-naringenin increased the T_m_ by ~2.7 °C. The positive shift in T_m_ caused by (2*S*)-hesperetin and (2*S*)-naringenin (Fig. S2) is comparable to the effect of 10 mM UDP, which causes a shift of 3 °C.

### Determination of IC_50_, K_D_ values, and the mechanism of inhibition of UGM inhibitors

**Figure 3:**
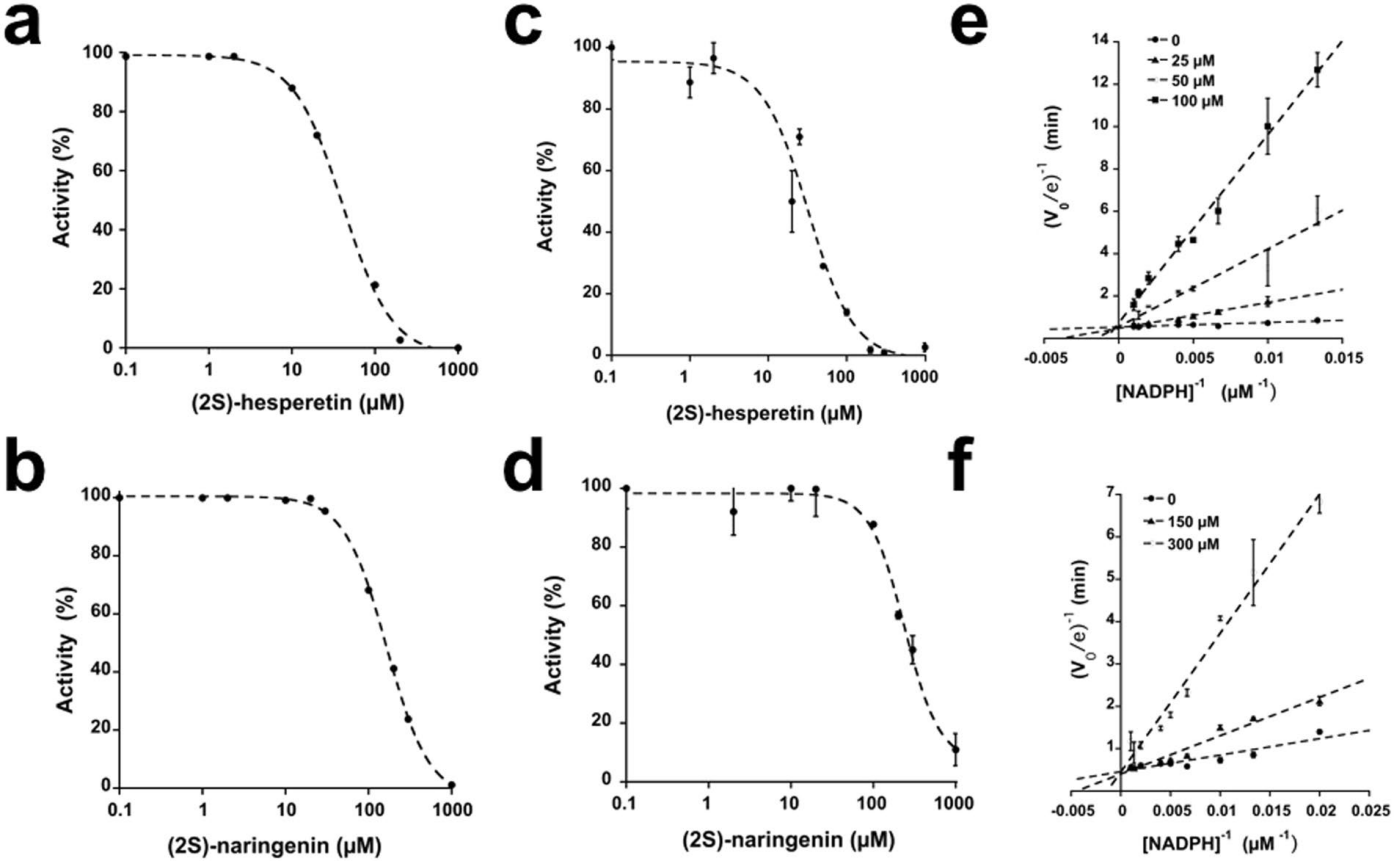
Characterization of UGM inhibitors. IC_50_ curves obtained with UPLC activity assay for **(a)** (2*S*)-hesperetin and **(b)** (2*S*)-naringenin. IC_50_ curves for **(c)** (2*S*)-hesperetin and **(d)** (2*S*)-naringenin obtained by following NADPH oxidation. Double reciprocal plots for the NADPH oxidation by UGM in the presence of **(e)** (2*S*)-hesperetin or **(f)** (2*S*)-naringenin.

**Figure 4:**
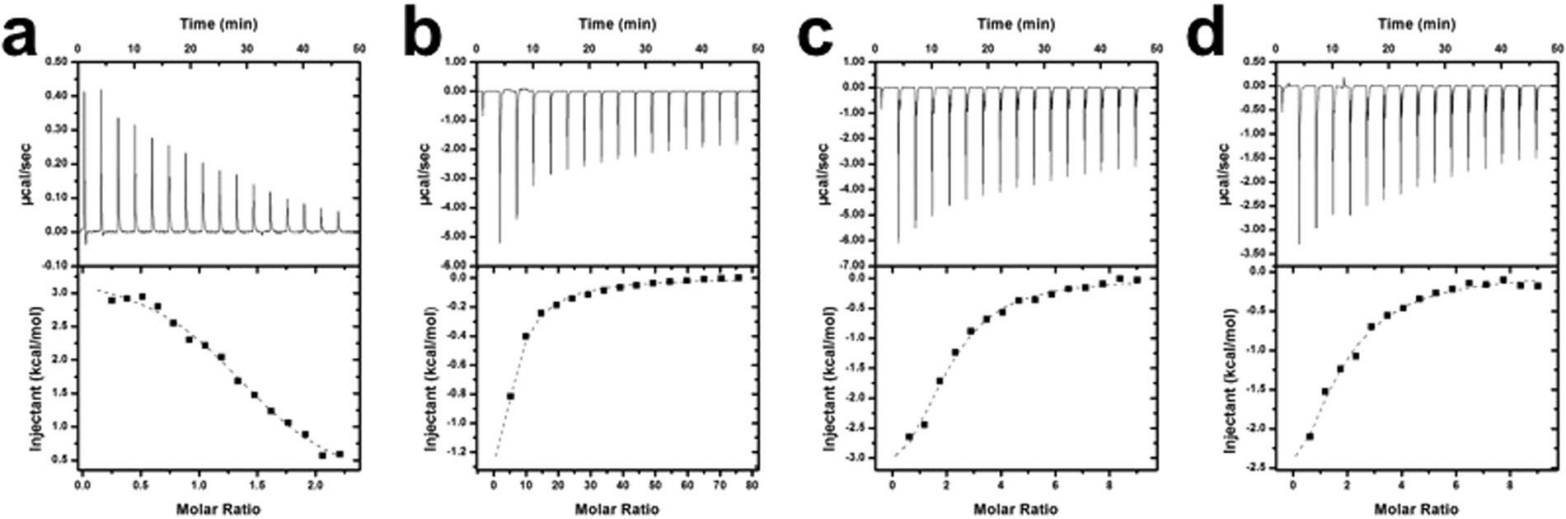
Representative ITC results and fitting curves for substrates and inhibitors binding to UGM. **(a)** UDP and **(b)** NADP^+^ are shown for reference. **(c)** (2*S*)-hesperetin and **(d)** (2*S*)-naringenin.

IC_50_ values were determined with the UPLC method using sodium dithionite as the reducing agent (Fig. 3a,b). Chrysin showed the highest potency followed by (2*S*)-hesperetin and (2*S*)-naringenin (Table 1). However, the IC_50_ curve of chrysin saturated below full enzyme inhibition (~35% activity, ~30 µM chrysin), and precipitated above 100 µM (with 2% DMSO). These behaviors agree with the reported mechanism that involves the formation of a colloidal aggregate by the compound, which precipitates as the concentration rises^26^. As expected, chrysin did not inhibit NADPH oxidation, nor was binding detected with ITC. The IC_50_ for (2*S*)-hesperetin and (2*S*)-naringenin was also determined by following NADPH oxidation by UGM in the absence of UDP-Gal*f* (Fig. 3c,d). It was found that (2*S*)-hesperetin and (2*S*)-naringenin inhibited NADPH oxidation. The type of inhibition by (2*S*)-hesperetin and (2*S*)-naringenin was assessed with the NADPH oxidation assay at different concentrations of inhibitor. (2*S*)-Hesperetin and (2*S*)-naringenin were competitive against NADPH. The K_i_ values for (2*S*)-hesperetin and (2*S*)-naringenin were 6 ± 1 µM and 74 ± 24 µM, respectively. The K_D_ for all ligands was determined by ITC measurement. The K_D_ values for (2*S*)-hesperetin and (2*S*)-naringenin were higher than that for NADP^+^ and lower than that for UDP (Fig. 4 and Table 2). Interestingly, the binding of NADP^+^, (2*S*)-naringenin, and (2*S*)-hesperetin was exothermic; the enthalpy driven binding of NADP^+^, (2*S*)-naringenin, and (2*S*)-hesperetin could indicate the formation of hydrogen bonds along with conformation changes. While the entropy driven binding of UDP could be related to hydrophobic interaction and water release.

**Table 1:** Characterization of UGM inhibitors identified by HTS of the Spectrum library using ADP-TAMRA. IC_50_ was determined with 20 µM UDP-Gal*f* (UPLC) and 10 mM sodium dithionite and for the NADPH oxidation was determined with 250 µM NADPH and no substrate.

Name	Chemical Structure	IC_50_ (µM)		Type of Inhibition	K_i_(µM)
		UPLC	NADPH oxidation		
(2*S*)-hesperetin		40 ± 3	32 ± 7	Competitive (NADPH)	6 ± 1
(2*S*)-naringenin		161 ± 11	233 ± 28	Competitive (NADPH)	74 ± 24
Chrysin		24 ± 4	>1 mM	—	—

**Table 2:** Thermodynamic parameters for the ligands UDP, NADP^+^ and inhibitors binding to *A. fumigatus* UGM.

Compound	∆G(kcal•mol^−1^)	∆H(kcal•mol^−1^)	T•∆S(kcal•mol^−1^)	*K*_D_(µM)
UDP	−7.1 ± 0.1	3.3 ± 0.1	10.5 ± 0.3	5.4 ± 0.8
NADP^+^	−4.6 ± 0.3	−13.0 ± 0.3	−8.4 ± 0.7	413 ± 24
(2*S*)-hesperetin	−6.1 ± 0.4	−4.1 ± 0.4	2.1 ± 0.2	31 ± 6
(2*S*)-naringenin	−5.7 ± 1.0	−4.8 ± 1.0	0.9 ± 0.3	65 ± 13

### Inhibition studies of (2*S*)-hesperetin and (2*S*)-naringenin derivatives

**Figure 5:**
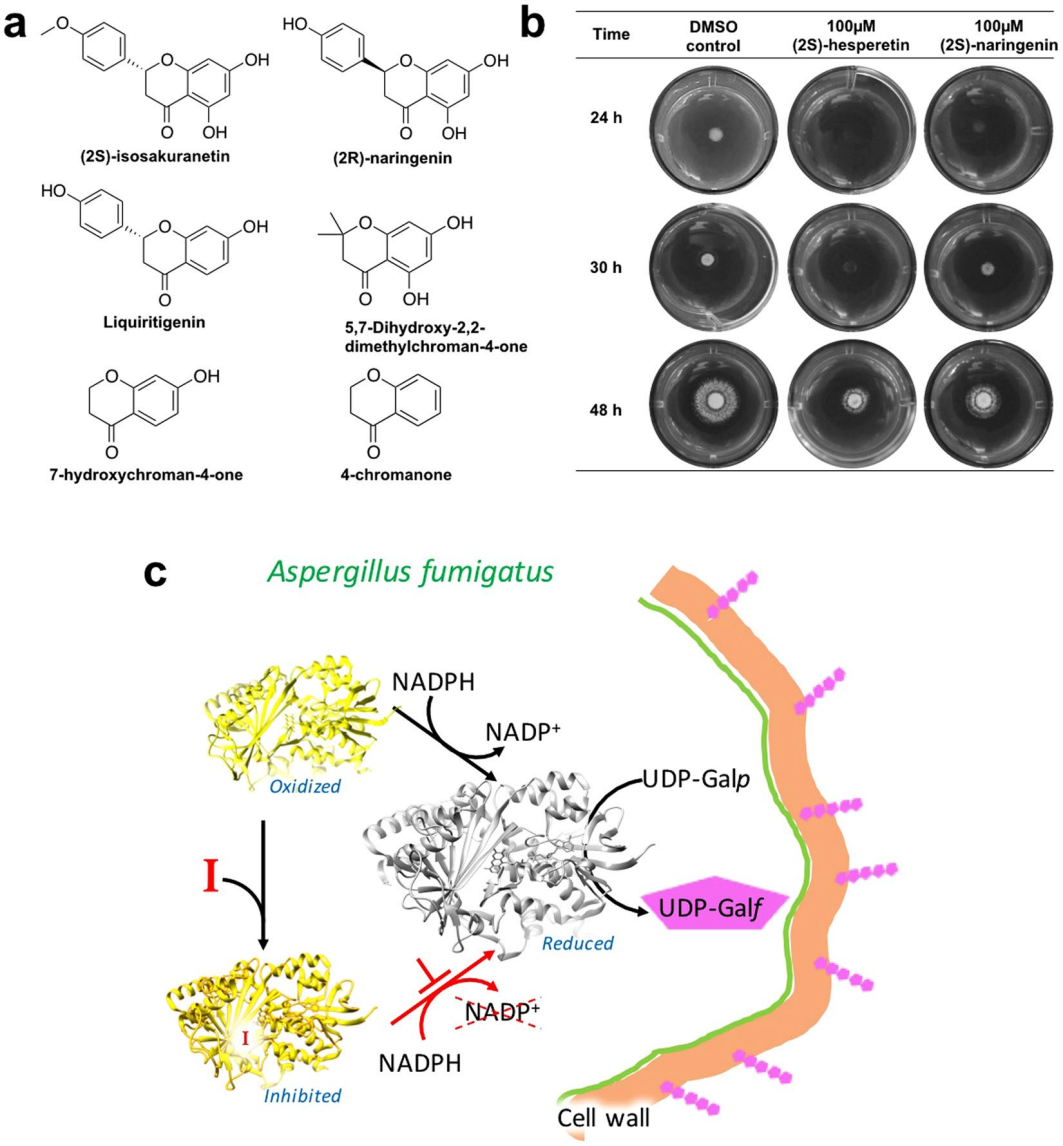
**(a)** Structures of the (2*S*)-hesperetin and (2*S*)-naringenin derivatives studied in this work. **(b)** Susceptibility of *A. fumigatus* to congo red (0.5 mg/mL) and 100 µM (2*S*)-hesperetin and (2*S*)-naringenin. **(c)** Graphical depiction of the proposed attenuation strategy against *A. fumigatus* virulence. Inhibition of UGM traps it in its oxidized, or inactive, state and leads to the abolishment of UDP-Gal*f* production and cell wall weakening.

To characterize the structural components important for inhibition, commercially available derivatives of (2*S*)-hesperetin and (2*S*)-naringenin were studied. The selected compounds included modification in the phenyl moiety and/or in the dihydroxrychroman moiety of (2*S*)-hesperetin as well as different hydroxychroman structures (Fig. 5a). (2*S*)-isosakuranetin was the only compound that inhibited the activity of UGM with an IC_50_ of 113 ± 9 µM (UPLC assay, Fig. S3) and was not competitive with NADPH. The IC_50_ for (2*S*)-isosakuranetin is comparable to the IC_50_ of (2*S*)-naringenin. (2*S*)-isosakuranetin increased the T_m_ of UGM by 1.3 ± 0.2 °C. This change in T_m_ is half the increase caused by (2*S*)-hesperetin and (2*S*)-naringenin (Fig. S2). No binding of (2*S*)-isosakuranetin to UGM was detected using ITC measurements. The structural difference between (2*S*)-hesperetin, (2*S*)-naringenin, and (2*S*)-isosakuranetin lies in the substituents of the phenyl group. In (2*S*)-hesperetin, the phenyl ring contains the 3-hydroxy-4-methoxy groups, while (2*S*)-naringenin and (2*S*)-isosakuranetin lack the 4-methoxy or the 3-hydroxy substituent, respectively. Modification in the 5,7-dihydroxrychroman-4-one moiety of (2*S*)-naringenin, specifically removing the 5-hydroxyl group of (2*S*)-naringenin, results in the compound liquiritigenin with no inhibitory effect against UGM. The *R* enantioniomer of naringenin and the dihydroxychroman derivatives showed no inhibition of UGM.

### Activity of UGM inhibitors in *A. fumigatus* cultures

We observed no* A. fumigatus* toxicity with (2*S*)-hesperetin and (2*S*)-naringenin at 1000 µM in PDA medium at 37 °C (Fig. S4). We performed combined susceptibility assays to congo red (0.5 and 1 mg/mL) with 100 µM (2*S*)-hesperetin and (2*S*)-naringenin. After 48 h of incubation at 37 °C we observed a growth decrease of ~50% and ~30% with (2*S*)-hesperetin and (2*S*)-naringenin respectively (Fig. 5b). When *A. fumigatus* was grown for 48 h at 50 °C in PDA with 1000 µM (2*S*)-hesperetin or (2*S*)-naringenin, we observed a delay in colony development of ~50% and ~30% respectively when compared with the reference (Fig. S5).

## Discussion

The cell wall of *A. fumigatus* is a complex and dynamic structure able to reprogram its composition to overcome genetic deficiencies and environmental stress^27^. Current drugs against invasive and systemic *A. fumigatus* infections include polyenes (*e.g*., amphotericin B), azoles (*e.g*., voriconazole), and echinocandins (*e.g*., caspofungin). Polyenes can bind to sterols and form pores in the cell membrane leading to increased membrane permeability. Azoles can block ergosterol biosynthesis causing an accumulation of lanosterol that reach toxicity levels. Echinocandins are the only antifungals that target the cell wall by inhibiting β-1,3-glucan synthase^27,28^.

During infection, cell walls containing galactofuranose polysaccharides, specifically galactomannan, play important roles in host immune recognition^11^. Secreted galactomannans in association with galactosaminogalactans are the major components of the biofilm mycelia found in aspergilomas and pulmonary lesions^8^. UGM is a key enzyme﻿ in the biosynthesis of galactofuran-containing molecules and is a virulence factor in *A. fumigatus*^12^. There are several research reports of inhibitors targeting the UDP-Gal*p* binding site of prokaryotic UGM. Recently, natural and synthetic flavonoids were reported as *M. tuberculosis* UGM inhibitors with a non-competitive mechanism of action^29^. Different approaches to developing UGM inhibitors have been reported. These includes a cell-free enzymatic synthesis of UDP-Gal*f* derivatives modified at C-5 and C-6, which prevent mycobacterial galactan polymerization^30^. To date, there are no reports of inhibitors that target the unique NADPH binding site of UGM.

In this work, we reported that (2*S*)-hesperetin competitively inhibits UGM in its oxidized state by binding to the NADPH site and that (2*S*)-hesperetin is able to inhibit the conversion of UDP-Gal*f* to UDP-Galp with an IC_50_ of 40 µM. The potency of (2*S*)-hesperetin is higher than the most recent report targeting the UDP-Gal*f* binding site of UGM from *M. tuberculosis* where, exo-glycans bearing both a sulfone and a phosphonate irreversibly inhibited UGM with an IC_50_ of 77 µM^31^. These two compounds, (2*S*)-hesperetin and (2*S*)-naringenin, are flavonones found in citrus (orange and grapefruit). (2*S*)-Hesperetin is known to be an anti-inflammatory and immunomodulatory agent^32^. (2*S*)-hesperetin has shown cytoprotective activity in models of acute liver toxicity^33^ and has a protective effect against H_2_O_2_-induced oxidative damage in ARPE-19 cells^34^. Nevertheless, it has been reported that (2*S*)-hesperetin and (2*S*)-naringenin have an inhibitory effect on UDP-glucuronosyltransferases^35^. Antimicrobial activities of (2*S*)-hesperetin include biofilm formation inhibition >70% against *S. aureus* strains, where the formation of biofilm by *S. aureus* is associated with the synthesis of an extracellular β-1,6-linked *N*-acetyl (succinyl) glucosamine polymer^36^.

(2*S*)-Hesperetin and (2*S*)-naringenin were shown to be competitive inhibitors against NADPH, suggesting that these compounds bind to the active site of UGM. Thus, these compounds can prevent activation of UGM by blocking NADPH binding. Furthermore, these compounds effectively block the mutase activity of UGM as inhibition of chemically reduced UGM was also observed. It is possible that binding of flavonoids prevents proper closing of the active site flaps and, thus, UDP binding^19,21^. The thermodynamic behavior of the interaction between UGM and (2*S*)-hesperetin and (2*S*)-naringenin resembles the binding of NADP^+^, which is exothermic and enthalpy-driven. In contrast, the binding of UDP is endothermic and entropy-driven. Binding enthalpies between (2*S*)-hesperetin and (2*S*)-naringenin are not significantly different. Even so, the IC_50_ and K_i_ values of (2*S*)-hesperetin are about 10 times lower than for (2*S*)-naringenin. The discrepancy between binding enthalpies and inhibition constants could be a consequence of enthalpy gain compensated by entropy loss resulting from strong hydrogen bonds between UGM and (2*S*)-hesperetin^37,38^ and, most likely, by the methoxy and hydroxy substituents of the phenyl ring of (2*S*)-hesperetin. (2*S*)-Hesperetin and (2*S*)-naringenin have a stabilizing effect on UGM, increasing the melting temperature. The increased stability is attributed to a tighter conformation resulting from the binding of a small molecule.

It has been reported that cell wall mutants are generally more sensitive to the anionic dye congo red, which interferes with the construction and stress response of the cell wall. The susceptibility to congo red is a response related to an increase in chitin deposition in the cell wall product of disturbed synthesis of β-1,3-glucan or β-1,6-glucan, mannosylation of mannoproteins, and glycosylphosphatidylinositol biosynthesis, among others^39^. We observed an increased susceptibility to congo red in the presence of (2*S*)-hesperetin and (2*S*)-naringenin that could be related to changes in the galactomannan biosynthesis induced by partial UGM inhibition. Future crystallography studies of UGM with (2*S*)-hesperetin and (2*S*)-naringenin will allow design and optimization of more potent inhibitors of eukarytoic UGMs.

## Materials and Methods

### Materials

Buffers, antibiotics, and bacterial growth media were obtained from Fisher Scientific (Pittsburg, PA) and Sigma-Aldrich (St. Louis, MO). Turbo BL21 (DE3) chemically competent cells were obtained from Genlantis (San Diego, CA). For protein purification, an AKTA prime system (GE Healthcare) was used along with IMAC columns (GE Healthcare). Acquity ultraperformance liquid chromatography (UPLC) and Amide (1.7 µm, 2.1 mm × 100 mm) analytical columns were obtained from Waters (Milford, MA). NADPH was obtained from EMD Biosciences (Billerica, MA). UDP-Gal*f* was synthesized as described previously^18^. The Spectrum Collection library was purchased from Microsource (Gaylordsville, CT). For the FP assay, non-binding, surface black 384-well plates were purchased from Greiner Bio-One (Monroe, NC) and samples were analyzed on a Spectramax M5 microplate spectrophotometer (Molecular Devices, Sunnyvale, CA). Differential scanning fluorimetry was performed in an RT-PCR (Applied Biosystems 7300) using 96-well RT-PCR plates (Microamp 4306737) with optical adhesive films (MicroAmp 431197971). ITC measurements were performed in an Auto-ITC 200 from Malvern Instruments (Malvern, United Kingdom) and analyzed using the Microcal Origin version 7.0 from OriginLab (Northampton, MA). (2*S*)-Hesperetin was purchased from TCI America (Portland, OR) and (2*S*)-naringenin and (2*S*)-isosakuranetin from Fisher Scientific (Asheville, NC). (2 *R*)-naringenin was purchased from Vitas-M Laboratory (Champaign, IL). 5,7-dihydroxy-2,2-dimethylchroman-4-one was purchased from Hit2Lead (San Diego, CA). 7-hydroxychroman-4-one, 4-chroman, and liquiritigenin were purchased from ArkPharm (Arlington Heights, IL). Dehydrated potato dextrose agar was from BD Difco (Franklin Lakes, NJ) and *Aspergillus fumigatus* strain 46645 was purchased from the American Type Culture Collection (ATCC, Manassas, VA).

### Expression and purification of *A. fumigatus* UGM

UGM was expressed in the vector pVP55A as reported previously^18^. Briefly, 6-L of terrific broth auto induction media containing 100 μg/ml ampicillin were inoculated with 8 mL of overnight culture of BL21 Turbo cells transformed with the vector pPV55A and inoculated at 37 °C until an O.D. of 3 was reached. Then, the temperature was dropped to 18 °C and cultures were incubated for 18 additional hours. Cells were harvested by centrifugation at 5000 *g* for 20 min at 4 °C. The final wet-cell pellet (75 g) was stored at −80 °C. For protein purification, the cell paste was suspended in 250 mL of buffer A (25 mM HEPES, 300 mM NaCl, 25 mM imidazole, pH 7.5) and incubated with 25 mg/mL of lysozyme, DNAse I, and RNAse for 45 min at 4 °C with constant stirring. The resulting solution was sonicated in an ice bath for 15 min at 70% amplitude at cycles of 5*S* ON and 10*S* OFF. The lysate was centrifuged at 45,000 *g* for 45 min and the supernatant was collected and loaded onto three in-tandem 5 mL HisTrap columns previously equilibrated with buffer A. After loading was completed, the columns were washed with buffer A until the absorbance at 280 nm returned to baseline levels. UGM was eluted with Buffer B (25 mM HEPES, 300 mM NaCl, 300 mM imidazole, pH 7.5). To remove the 8× His-tag, the protein was dialyzed in the presence of 8× His-tobacco etch virus (8× His-Tev) protease (1:20 ratio) at 4 °C with slow stirring in buffer C (25 mM HEPES, 300 mM NaCl, pH 7.5). The dialyzed sample was loaded back onto the IMAC, previously equilibrated with buffer C, and the flow-through containing UGM was collected. The buffer of the purified UGM was exchanged to 25 mM HEPES with 100 mM NaCl, flash frozen, and stored at −80 °C until use.

### Binding of ADP-TAMRA to UGM and competition with NADP^+^ and UDP

Our group previously developed a binding assay to identify inhibitors of flavoenzymes that use NADP(H) as substrate. Since UGM also used NADPH for reduction, we determined whether this compound bound to UGM. This was done by taking the ADP-TAMRA^22^ chromophore to a final concentration of 30 nM in 25 mM HEPES buffer, pH 7.5, mixed with various concentrations of UGM (0–300 µM, based on Bradford). To determine if the chromophore was binding to the active site of UGM, the competitive binding of UDP (0–1 mM) and NADP^+^ (0–10 mM) was calculated by measuring the decrease in anisotropy of the UGM (6.7 µM) - ADP-TAMRA (30 nM) complex. At each concentration of ligand, the solution was incubated for 10 min at room temperature. The sample was excited at 544 nm and the emission recorded at 584 nm using a wavelength cutoff of 570 nm.

### High-throughput screening

High-throughput screening of the Spectrum Collection library using the FP assay with the ADP-TAMRA^22^ probe was performed at the Virginia Tech Center for Drug Discovery Screening Laboratory. 384 well plates (Greiner 784900) were used and the reaction volume was 15 µL using 25 mM HEPES buffer (pH 7.0). For the screening, 30 nM of ADP-TAMRA with 6.7 µM UGM (based on the Bradford assay) and 20 µM of library compound were used, with a final DMSO concentration of 2%. Fluorescence polarization measurements were performed with excitation at 544 nm and emission at 584 nm, using a wavelength cutoff of 570 nm. Anisotropy measurements were performed after 30 and 120 minutes of incubation at 25 °C. Anisotropy values were normalized to the values obtained in the negative control sample: UGM, ADP-TAMRA, and DMSO (high anisotropy values). The positive control consisted of ADP-TAMRA and DMSO (low anisotropy values). The Z prime (Z′) value was determined using equation (1) where, σ_N,P_ and μ_N,P_ represent the standard deviation and the mean value the of the negative and positive controls respectively.

1 $$Z^{\prime} =1-\frac{3({\sigma }_{N}+{\sigma }_{P})}{|{\mu }_{N}-{\mu }_{P}|}$$

### Hydrogen peroxide quantification assay

To identify compounds that oxidize NADPH, we screened the formation of hydrogen peroxide (H_2_O_2_) in the absence of UGM using the xylenol orange assay as previously reported^40^. The assay was performed in 384 well plates (Greiner, 781162) and the reaction volume was 15 μL and consisted of 50 mM HEPES buffer (pH 7.5) containing 200 mM NaCl, 500 µM NADPH, and 200 µM of compound (dissolved in DMSO). The final DMSO concentration in the reaction mix was 2%. Controls contained 2% DMSO instead of compound. The reaction was started by the addition of NADPH and allowed to proceed at room temperature in the dark. After 45 min, the reaction was quenched with 85 µL of a freshly made solution of 200 mM sorbitol, 250 µM xylenol orange, 500 µM ammonium ferrous sulfate, and 25 mM sulfuric acid. After 10 minutes of incubation, the absorbance was measured at 595 nm. The concentration of H_2_O_2_ in samples was determined by comparison with a predetermined H_2_O_2_ standard curve containing 2% DMSO.

### ThermoFAD assays

The melting temperature^25^ of UGM was determined by measuring the FAD fluorescence as a function of temperature (20–90 °C, 1 °C step) following the reported ThermoFAD procedure^41^. The reaction mix consisted of 20 µL of 1 mg/mL UGM, 2% DMSO in 50 mM potassium phosphate buffer, pH 7.0. As a reference and positive control, the T_m_ was calculated in the presence DMSO and UDP (10 mM). Different concentrations of inhibitors were added in the range of 0–1 mM at a final DMSO concentration of 2% in the above-mentioned buffer. All experiments were done in triplicate.

### Activity assay, IC_50_, and K_i_ determination

The activity of UGM and IC_50_ was determined by monitoring the formation of UDP-Gal*p* from UDP-Gal*f* at 262 nm in a UPLC system. A 30 µL reaction containing 25 mM HEPES buffer with 125 mM NaCl, 20 µM UDP-Gal*f*, 10 mM sodium dithionite, 10 nM UGM (concentration based on FAD), 2% DMSO, and inhibitor (0–1 mM) were incubated at 25 °C for 3 min. Protein and inhibitor were pre-incubated for 5 min at 25 °C in reaction buffer (25 mM HEPES containing 125 mM NaCl) and the reaction was started with the addition of 3 µL of 200 µM UDP-Gal*f*. The reaction was quenched with the addition of 70 µL acetonitrile. After removing the protein by centrifugation, 5 µL of sample was injected into an Amide column (Acquity UPLC BEH Amide, 1.7 µm, 2.1 mm × 100 mm) at 45 °C. The UPLC column was pre-equilibrated with 75% acetonitrile and 25% 27 mM aqueous HPLC-grade potassium phosphate (pH 4.5). Samples were run at a flow rate of 0.5 mL/min for 5 min. IC_50_ was also determined by following NADPH oxidation at 340 nm. The reaction mix consisted of 250 µM NADPH, 25 mM HEPES with 125 mM NaCl, 2.5 µM UGM, 2% DMSO, and inhibitor (0–1 mM). Data was analyzed using KaleidaGraph software. The type of inhibition was assessed by spectrophotometrically (340 nm) recording the oxidation of various concentrations of NADPH (0–1 mM) in 25 mM HEPES containing 125 mM NaCl with 2.5 µM UGM (based on FAD) at different concentrations of inhibitor with 2% DMSO in the absence of UDP-Gal*f*. Global fitting was performed with Graphpad Prims 6 software (La Jolla, CA).

### ITC measurement

Binding of inhibitors to UGM was measured with an Auto-ITC 200 ITC instrument. UGM was dialyzed in 50 mM potassium phosphate buffer, pH 7.0, for 2 h at 4 °C. In all cases, ligands were dissolved in dialysis buffer and protein and ligand were filtered with a 0.2 µM membrane prior to loading. UGM concentration was 46 µM in all titrations and the concentrations of NADP^+^ and UDP in the syringe were 16.8 mM and 400 µM, respectively. Inhibitor concentration in the syringe was 2 mM (2% DMSO), except for chrysin where the concentration was 600 µM (4% DMSO). Titration was performed in 16 injections of 2.5 µL with spacing of 180*S* at 25 °C with continuous stirring at 750 rpm. Thermodynamic parameters were determined from ITC results using Microcal Origin version 7.0 (OriginLab) with the “ITC custom” add-on installed.

### Effect of (2*S*)-hesperetin and (2*S*)-naringenin on *A. fumigatus* growth

Spores of *A. fumigatus* from ATCC were germinated in potato dextrose agar (PDA). Spores were harvested using a sterile plastic scraper from 3 day old cultures grown at 37 °C and suspended in phosphate saline buffer containing 0.1% Tween-20. Inhibitor effect on cell growth was tested in 12-well plates (Falcon, 353043) containing 700 µL agar at a final DMSO concentration of 1% and spot inoculated with 1 × 10^3^ spores. For the toxicity studies, complete medium PDA containing 100–1000 µM of inhibitor was spot inoculated with 1 × 10^3^ spores and incubated at 37 °C. A control was inoculated into PDA with DMSO. Inhibitor or DMSO was mixed with the PDA agar before solidifying. For congo red susceptibly assays, inhibitors at 100 µM concentration were added to aspergillus minimum media (AMM)^42^ containing congo red (1.0 and 0.5 mg/mL) before the agar jellified. The working concentrations of congo red^39^ were determined by a serial dilution method in a 12-well plate inoculated with 1 × 10^3^ spores and grown at 37 °C for 48 h. As the negative control, inoculated agar containing either congo red or 100 µM inhibitor were employed. For the thermotolerance studies, PDA medium containing 1000 µM of inhibitor was spot inoculated with 1 × 10^3^ and 1 × 10^5^ spores and incubated at 50 °C for 48 h. As control, agar with DMSO was employed.

## Electronic supplementary material

Supplementary Information
