# Author Correction: Identification of *Aspergillus fumigatus* UDP-Galactopyranose Mutase Inhibitors

**DOI:** 10.1038/s41598-018-22958-7

**Published:** 2018-03-13

**Authors:** Julia S. Martin del Campo, Meital Eckshtain-Levi, Nancy J. Vogelaar, Pablo Sobrado

**Affiliations:** 1 Department of Biochemistry, Virginia Tech, Blacksburg, VA 24061 USA; 2 Virginia Tech Center for Drug Discovery, Virginia Tech, Blacksburg, VA 24061 USA

Correction to: *Scientific Reports* 10.1038/s41598-017-11022-5, published online 07 September 2017

The Acknowledgements section in this Article is incomplete.

“This work was supported by a National Institutes of Health Grant GM094469 and funds from the Fralin Life Science Institute. We are grateful to Dr. Karina Kizjakina for synthesizing and purifying the UDP-Galf.”

should read:

“This work was supported by a National Institutes of Health Grant GM094469, Fralin Life Science Institute, and the Virginia Tech Open Access Subvention Fund. We are grateful to Dr. Karina Kizjakina for synthesizing and purifying the UDP-Galf.”

