# Supplementary material for: Identification of Aspergillus fumigatus UDP-Galactopyranose Mutase Inhibitors
Source: Sci Rep. 2017 Sep 7;7:10836. doi: 10.1038/s41598-017-11022-5 (PMC5589893; doi:10.1038/s41598-017-11022-5)
Supplement: Supplementary file 1 — Supplementary Information [file 41598_2017_11022_MOESM1_ESM.pdf]

Identification of *Aspergillus fumigatus* UDP-galactopyranose mutase inhibitors

(Supporting Data)

Julia S. Martín del Campo,[1] Meital Eckshtain-Levi[1], Nancy Vogelaar,[2] and Pablo Sobrado[1,2 *]

[1] Department of Biochemistry, Virginia Tech, Blacksburg, VA, 24061

[2]Virginia Tech Center for Drug Discovery, Virginia Tech, Blacksburg, VA, 24061

*Corresponding author

E-mail: psobrado@vt.edu (PS)

Table S1. Summary and classification of hits purchased from independent companies. Anisotropy readings from screening were normalized to negative control (protein and 30 nM ADP-TAMRA). Inhibition against UGM was assayed at 20 and 200 μM with 20 μM UDP-Gal*f* and 10 mM sodium dithionite using the UPLC method.

Family	Name	Normalized anisotropy (%)	Inhibition[a] > 50 %	Concentration effect[b]
Flavonoids	Hesperetin	17	+	+
	Naringenin	23	+	+
	Chrysin	48	+	+
	Kaempferol[c]	38	+	+
Non-Flavonoids	Nicorandil	27	-	-
	Diclazuril	37	+	+
	Diffractaic acid	37	-	-
	Febuxostat	39	+	+
	Olsalazine sodium	48	-	-
	Ellagic acid[d]	50	-	-
	Aurin tricarboxylic acid	14	-	-
	Nimesulide	28	-	-
	Suramin hexasodium	41	-	-

[a] Inhibition of product formation was determined by UPLC with 200 μM of compound.
[b] Inhibition of product formation at 200 μM compound concentration > inhibition at 20 μM compound concentration.
[c] The K_D curve of kaempferol (30 nM ADP-TAMRA) plateaued at around 50 % anisotropy. Similar behavior was observed on the IC_{50} curve, that plateaued at around 70 % enzyme activity.
[d] Due to low solubility, inhibition assays were performed with 6 and 12 μM ellagic acid with 2% DMSO.

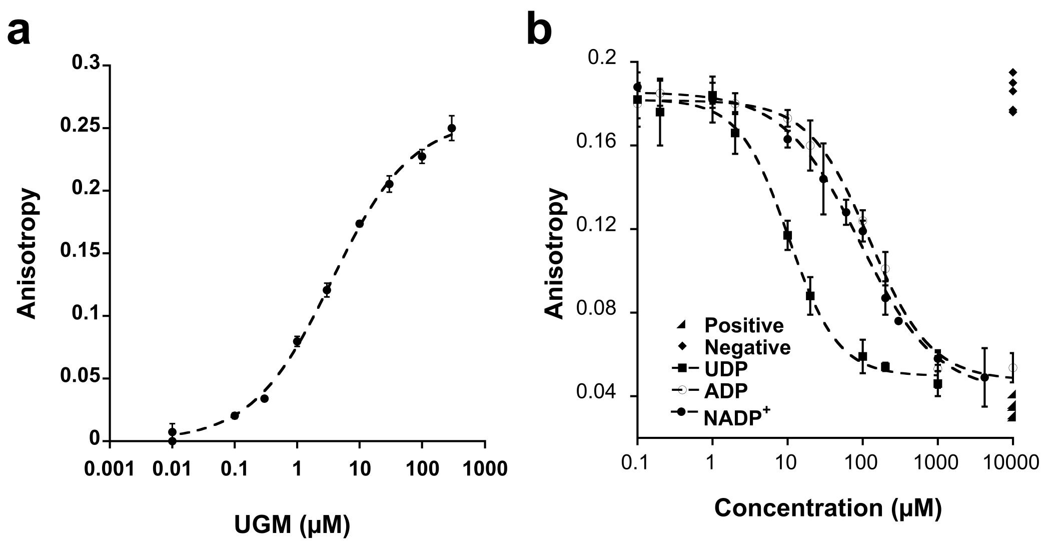

Fig. S1. (a) Binding isotherm of ADP-TAMRA (30 nM) to UGM. **(b)** Competitive binding of UDP, ADP, and NADP$^+$. The K_D values are 10 ± 1 µM, 124 ± 16 µM, and 92 ± 13 µM, respectively. Negative control samples consisted of UGM and ADP-TAMRA (30 nM) with no other ligand (complex with high anisotropy value). The positive control contained only ADP-TAMRA (free chromophore with low anisotropy value).

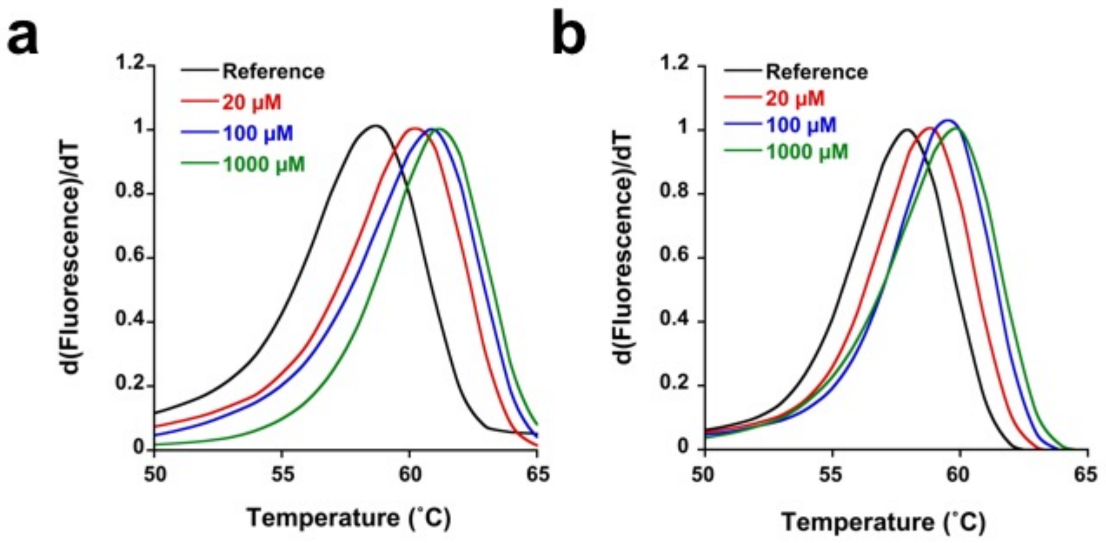

Fig. S2. Melting curves obtained with the ThermoFAD method for UGM at different concentrations of **(a)** (2*S*)-hesperetin and **(b)** (2*S*)-naringenin. As a reference, UGM with 2% DMSO was used.

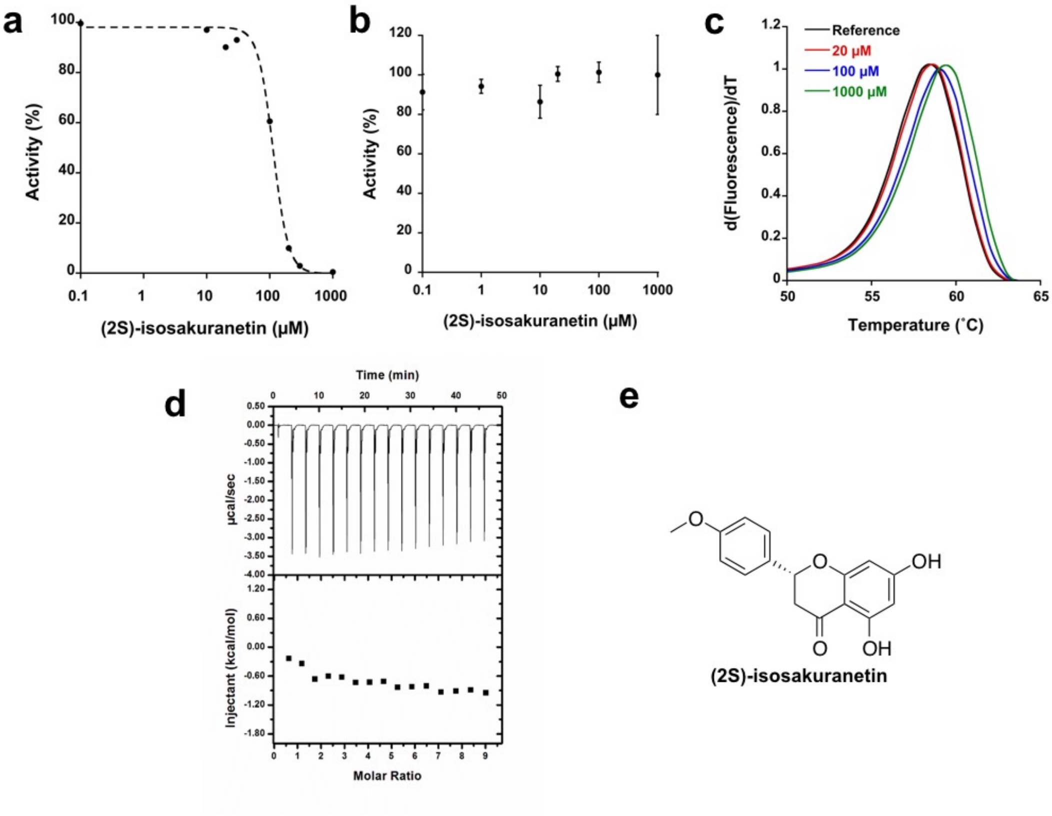

Fig. S3. Characterization of (2*S*)-isosakuranetin. **(a)** IC_{50} curve obtained using the UPLC activity assay. **(b)** NADPH (250 μM) oxidation by UGM at various concentrations of (2*S*)-isosakuranetin. **(c)** Melting curves of UGM in the presence of 20, 100, and 1000 μM (2*S*)-isosakuranetin and 2 % DMSO. **(d)** ITC results; no binding of (2*S*)-isosakuranetin was observed. **(e)** Structure of (2*S*)-isosakuranetin.

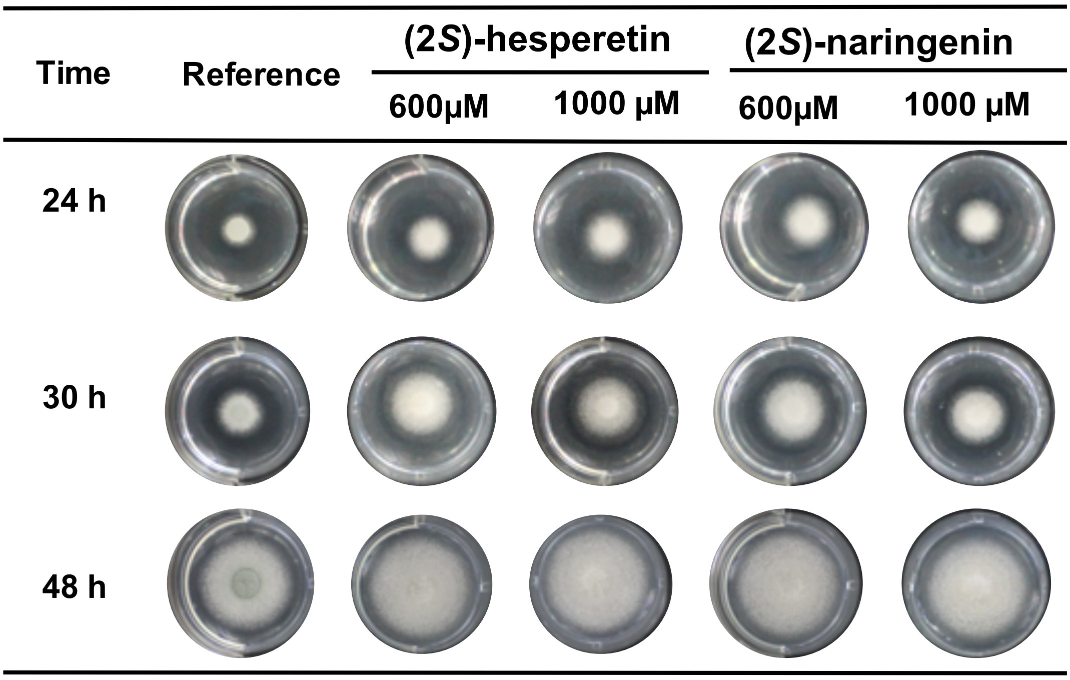

Fig. S4. *A. fumigatus* in PDA medium grown at 37 ˚C in the presence of 600 and 1000 µM (2*S*)-hesperetin or (2*S*)-naringenin. These experiments show that under optimal growth conditions these compounds are not toxic.

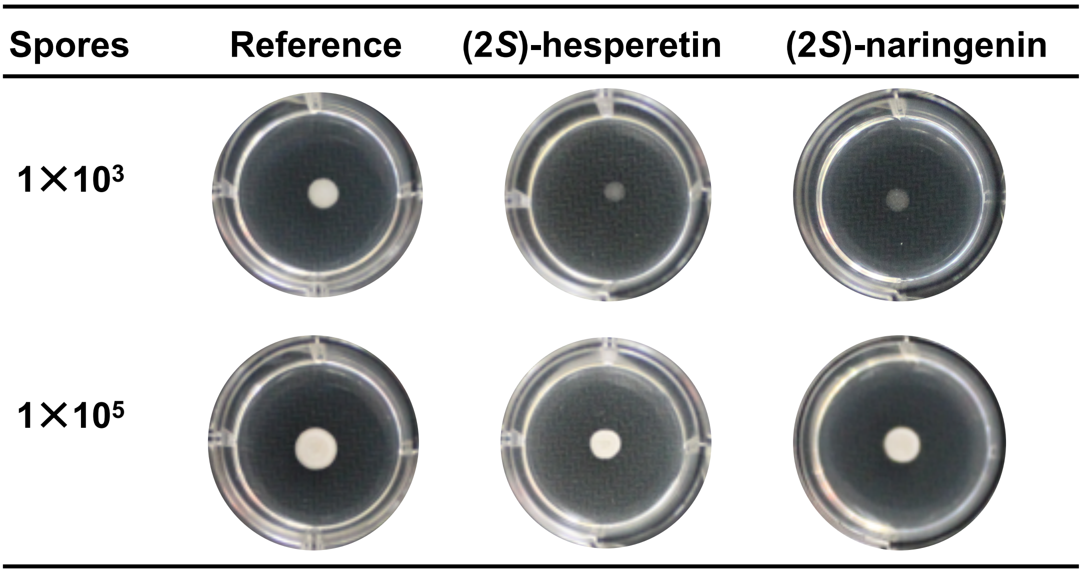

Fig. S5. Cultures of *A. fumigatus* in PDA medium containing 1000 µM (2*S*)-hesperetin or (2*S*)-naringenin. Agar was spot inoculated with 1×10^3 or 1×10^5 spores and incubated at 50 ˚C for 48 h. The reference consisted of PDA with DMSO.
